# Effect of low birth weight on childhood asthma: a meta-analysis

**DOI:** 10.1186/1471-2431-14-275

**Published:** 2014-10-23

**Authors:** Xue-Feng Xu, Ying-Jun Li, Yuan-Jian Sheng, Jin-Ling Liu, Lan-Fang Tang, Zhi-Min Chen

**Affiliations:** Department of Pulmonology, The Children’s Hospital, Zhejiang University School of Medicine, Hangzhou, 310003 China; Department of Epidemiology and Health Statistics, School of Public Health, Zhejiang University, Hangzhou, China

**Keywords:** Birth weight, Childhood asthma, Meta-analysis, Systematic review

## Abstract

**Background:**

Low birth weight is strongly correlated with an increased risk of adult diseases. Additionally, low birth weight might be a risk factor for asthma later in life.

**Methods:**

A systematic literature search of the PubMed database from 1966 to November 2013 was conducted. The criteria for inclusion of papers were as follows: case–control or cohort studies; the odds ratio (OR) or risk ratio (RR) estimates with the corresponding 95% confidence intervals (CIs) were presented, or there were sufficient data for calculation; and studies were published in English up to October 2013. Random-effect and fixed-effect meta-analyses, meta-regression, and cumulative meta-analysis were conducted.

**Results:**

Thirteen cohort studies and 1,105,703 subjects were included. The overall pooled RRs (95% CIs) of asthma risk for low birth weight were 1.162 (fixed-effects model, 95% CI, 1.128–1.197) and 1.152 (random-effects model, 95% CI, 1.082–1.222). In stratified analyses, the effect of low birth weight on childhood asthma was strong, particularly in studies conducted in Europe, those with a small sample size, and those published recently. A meta-regression analysis did not find significant determinants.

**Conclusions:**

This meta-analysis shows that low birth weight significantly increases the risk of childhood asthma.

## Background

Asthma is a serious global health problem. Worldwide, an estimated 300 million people are affected by asthma. The global prevalence of asthma ranges from 1 to 21% of the population in different countries [1]. The prevalence of asthma is increasing in most countries, especially among children [2]. This increase may be closely associated with exposure to allergens (particularly pollens, molds, dust, and pet dander), tobacco smoke, exercise, air pollutants, and respiratory infections, but has yet to be fully explained [3]. Furthermore, children with low birth weight are prone to developing decreased respiratory function and having an increased risk of chronic respiratory symptoms during childhood [4]. Previous studies have shown that some prenatal or perinatal adverse factors could be associated with the development of asthma later in life [4–6].

The fetal origins of adult disease hypothesis proposes that fetal adaptation to an adverse intrauterine environment could lead to permanent changes in individual physiology and metabolism [7]. There is evidence that lower birth weight is strongly correlated with an increased risk of adult diseases, such as type 2 diabetes mellitus, hypertension, and cardiovascular disease. Additionally, impaired fetal growth is a risk factor for asthma later in life [8], especially in children with a very low birth weight [9]. However, previous studies on the relation between low birth weight and childhood asthma have provided controversial results. To the best of our knowledge, there are no previous systematic reviews that have estimated the overall effect of low birth weight on the risk of childhood asthma. Therefore, we conducted a systematic review and a meta-analysis of the existing evidence on the relation between low birth weight and the risk of childhood asthma.

## Methods

### Search strategy and inclusion criteria

We performed a systematic literature search of the PubMed database from 1966 to November 2013 using the following search command: birth weight and “asthma or wheezing or wheeze”. The abstracts identified by this search were screened to eliminate the obviously irrelevant abstracts (such as reviews, systemic reviews, and case reports). The criteria for paper inclusion were as follows: (i) case–control or cohort studies that focused on the association of low birth weight with childhood asthma (less than 16 years old); (ii) studies that presented the odds ratio (OR) or risk ratio (RR) estimates with the corresponding 95% confidence intervals (CIs), or sufficient data for calculation; (iii) normal birth weight as the reference category; and (iv) studies published in English up to October 2013. All publications providing sufficient information regarding the relation between low birth weight and childhood asthma were included, irrespective of whether this issue was their primary or secondary objective. We excluded studies that used asthma-like symptoms or other manifestations of respiratory impairment as an outcome, such as wheezing.

The outcome of interest was childhood asthma. The definition of asthma was based on a physician’s diagnosis, or a history of asthma reported by their parents. Our primary predictor variable was low birth weight, which was defined as a birth weight of less than 2500 g. For any study to be included in the present study, its outcome and predictor definitions had to be consistent or adaptable with our definitions. Both outcome and predictor variables were dichotomous.

### Data extraction

Four investigators independently carried out data extraction of the following items: authors, publication year, study design, country, population age, diagnosis of asthma, adjustment for confounding (family history, air pollution, sex, gestation age, smoking, and caesarean etc.), and effect size and corresponding estimates with 95% CIs. Two reviewers completed the quality assessment independently. A set of structured criteria modified from previous studies (Newcastle–Ottawa scale for cohort study) were used to complete the quality assessment of publications. A higher score indicates higher quality. In the case of disagreement of extracted data, discrepancies were resolved by discussion and achieved consensus.

### Statistical analysis

We abstracted the multivariate-adjusted risk estimates (OR or RR). The unadjusted risk estimates were calculated using original data when the adjusted was unavailable. The CIs were transformed to a log scale and then the standard error was calculated. A pooled summary relative risk was calculated according to the fixed-effects model and a random-effects model. Statistical heterogeneity was assessed with the I-squared (I^2^) value, which represents the percentage of total variation across different studies due to heterogeneity rather than chance. I^2^ values of 25%, 50%, and 75% have been related to low, moderate, and high heterogeneity, respectively [10]. A random-effects model was applied when there was notable heterogeneity (I^2^ index ≥50%), and otherwise, the fixed-effects model was used. Subgroup analyses were stratified by geographic area and adjusted for the major confounder. Meta-regression analysis was used to investigate the potential sources of heterogeneity. Cumulative meta-analysis in the order of publication year was conducted to check homogeneity of the results. Publication bias was assessed by using the funnel plot, the rank correlation test, and the graphical test [11–13]. All statistical analyses were carried out by STATA version 12.0 (Stata Corp., College Station, TX).

## Results

### Characteristics of included studies

**Figure 1:**
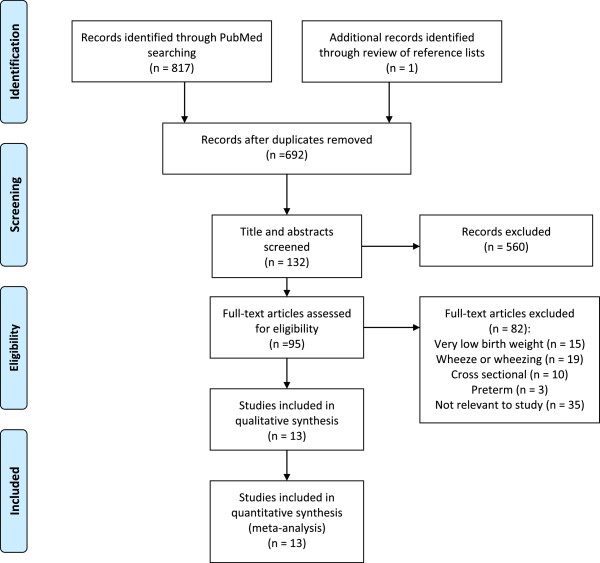
**Flow diagram for the selection of studies: article search strategy results.**

A description of the search process is shown in Figure 1. Of the 692 papers found by the search, 13 cohort studies met the inclusion criteria and were included [14–26]. Among these, only five studies provided the adjusted effect estimates of low birth weight versus childhood asthma [14–16, 22, 23]. Of the 13 cohort studies selected, 11 were published in 2000–2013, and two were published in 1997 and 1998. The majority of the cohorts (n = 6) were in Europe (Denmark, Finland, Germany, Sweden, and the United Kingdom) [14, 16, 20, 21, 24, 26]. Four cohorts were studied in North America (US and Canada) [19, 22, 23, 25], one in Oceania (New Zealand) [17], one in South America (Brazil) [15], and one in Asia (Taiwan) [18]. Detailed characteristics of the studies that were included are shown in Table 1.

**Table 1:** **Characteristics of the studies included in the analysis**

Study (reference)	Country of study, n.	Primary exposure measure (birth weight, g)	Effect size (95% CI)	Age at follow up (y)	Quality score	Adjusted
Davidson [16]	England, n = 248612	1000-2999 vs 3000-3999	OR 1.2 (1.1-1.3)	2-10	8.0	Yes
Chatkin [15]	Brazil, n = 981	< 2500 vs >2500	RR 1.32 (0.8-1.96)	4-5	5.5	Yes
Nepomnyaschy [19]	United States, n = 1803	< 2500 vs >2500	RR 1.57 (1.23-1.99)	< 3	6.0	No
Fergusson [17]	New Zealand, n = 798	< 2500 vs 2500-4000	RR 0.97 (0.6-1.58)	16	5.5	No
Lu [18]	Taiwan, n = 69979	< 3000 vs 3000-4000	RR 1.17 (1.09-1.27)	13-15	7.0	No
Yuan [26]	Denmark, n = 10190	< 2500 vs 2500-4499	RR 0.9 (0.43-1.89)	< 12	6.5	No
Wjst [24]	German, n = 2337	< 2500 vs >2500	RR 1.47 (0.64-3.36)	5-14	5.0	No
Remes [21]	Finland, n = 4141	≤ 2490 vs 2500-4190	RR 1.31 (0.85-2)	16	7.0	No
Yang [25]	United States, n = 3933	< 2500 vs >2500	RR 1.25 (0.73-2.15)	6-7	6.5	No
Rasanen [20]	Finland, n = 4502	< 2500 vs >2500	RR 0.97 (0.71-1.31)	16	6.0	No
To [23]	Canada, n = 671402	< 2500 vs 2500-4500	RR 1.18 (1.13-1.22)	6	8.5	Yes
Sin [22]	Canada, n = 83595	< 2500 vs 2500-4500	RR 1 (0.9-1.11)	10	7.5	Yes
Bjerg [14]	Sweden, n = 3430	< 2500 vs >2500	OR 2.6 (1.2-5.4)	7-8	6.0	Yes

### Exposure, outcome, and effect measures

Eleven studies used the definition of low birth weight compatible with our criteria (<2500 g). Birth weight in the other two studies was less than 3000 g [16, 18]. General variance-based methods using CIs were applied to combine the results according to birth weight. The definition of asthma was based on a physician’s diagnosis. Children with wheeze or wheezing were excluded.

Two studies reported data for the effect of low birth weight on the risk of future asthma as adjusted ORs [14, 16]. These studies used multivariate logistic regression to obtain results. The other three studies presented adjusted RRs. The rest of the eight studies presented prevalence based on birth weight. Prevalence was converted into crude RRs, and ORs and RRs were treated as comparable measures of effect (RR). The ranges of ORs and RRs varied from 0.9 to 2.6 (Table 1).

### Categorical meta-analysis for low birth weight and childhood asthma

**Figure 2:**
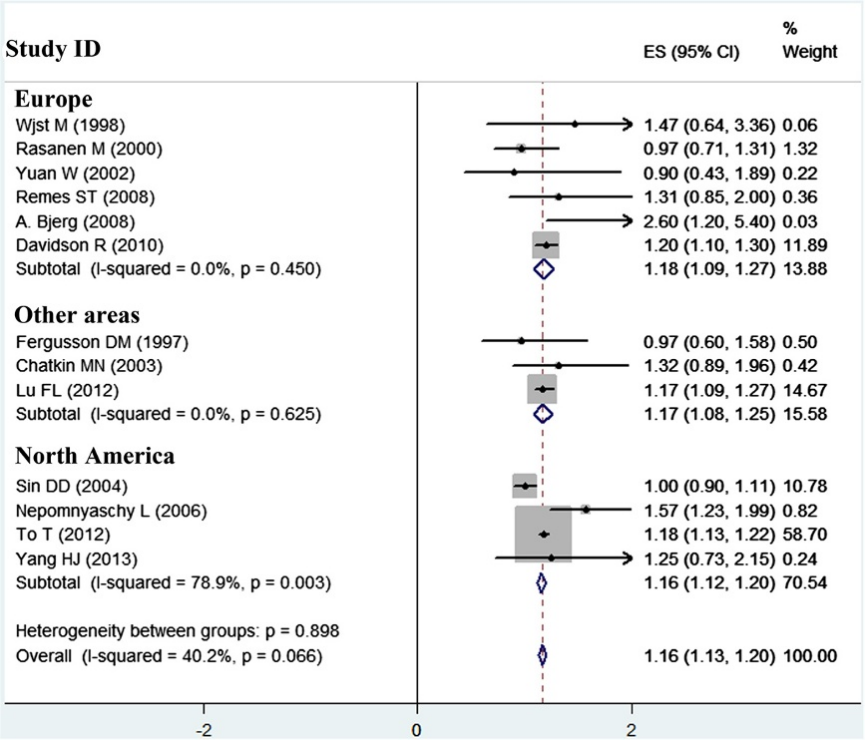
**Meta-analysis of the effect of low birth weight on childhood asthma, and forest plots stratified by geographic area.**

**Figure 3:**
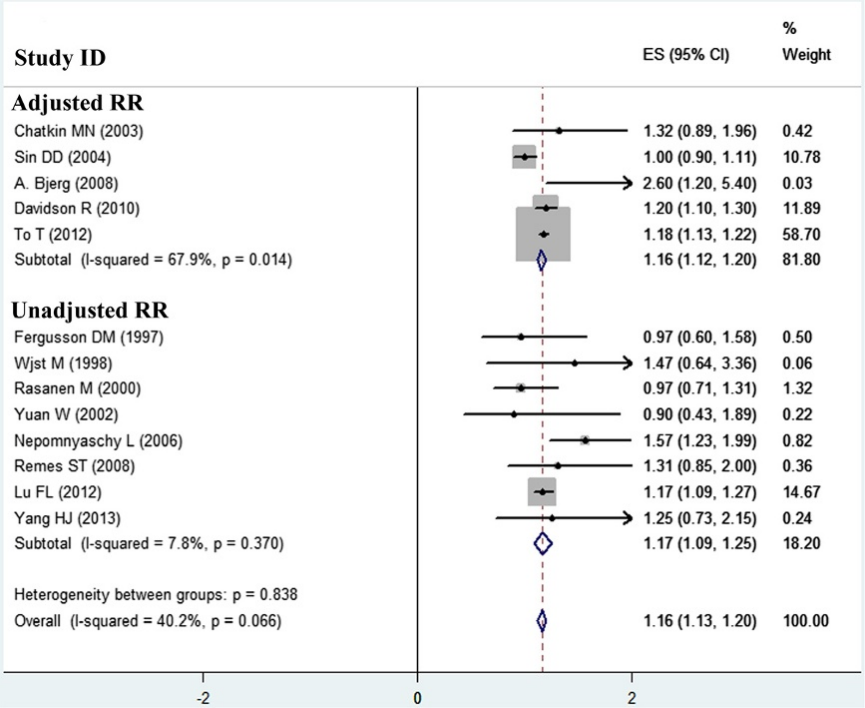
**Forest plot of the effect of low birth weight on childhood asthma stratified by adjusted and unadjusted RRs.**

Five of the 13 estimates were directly from the original studies, and eight were transformed from the information available to comply with our categorization used in the meta-analysis. Figures 2 and 3 show forest plots, which provide study-specific and pooled RRs (95% CIs), of development of asthma for low birth weight. When compared with normal birth weight, the pooled RRs from the fixed-effects and random-effects models were 1.162 (95% CI, 1.128–1.197) and 1.152 (95%, 1.082–1.222) for low birth weight (I^2^ = 40.2%, P = 0.066), respectively. Both models showed similar summary effect estimates, indicating a significantly increased risk of asthma in relation to low birth weight. Table 2 shows the pooled RRs (95% CIs) among different subgroups stratified by five main characteristics of the studies. The subgroup analyses showed an even greater effect estimate in the six studies conducted in Europe compared with other geographic areas. Additionally, subgroup analyses showed a greater effect estimate in the eight studies with a small sample size (<5000 subjects) compared with those with a larger sample size, and a greater effect estimate in the seven recently published studies compared with those in the older six studies. The effect estimates stratified by study population age and adjusted RRs were similar to the overall pooled RRs.

**Table 2:** **Pooled and subgroup analyses**

Stratification	Number of study	Model		Heterogeneity statistics	
		Fixed-effects model (RR, 95% CI)	Random-effects model (RR, 95% CI)	I ^2^	P value
Overall	13	1.162 (1.128-1.197)	1.152 (1.082-1.222)	40.2%	0.066
Geographic area					
Europe	6	1.18 (1.09-1.27)	1.18 (1.09-1.27)	0%	0.450
North America	4	1.16 (1.12-1.20)	1.17 (1.00-1.34)	78.9%	0.003
Other	3	1.17 (1.08-1.25)	1.17 (1.08-1.25)	0%	
Study population size					
< 5000	8	1.21 (1.03-1.39)	1.23 (1.20-1.22)	21.7%	0.257
≥ 5000	5	1.16 (1.12-1.20)	1.14 (1.07-1.21)	63.1%	0.029
Publication year					
1997-2005	6	1.01 (0.91-1.10)	1.01 (0.91-1.10)	0%	0.857
2006-2013	7	1.19 (1.15-1.22)	1.19 (1.15-1.23)	2.3%	0.408
Adjusted RR					
Yes	5	1.16 (1.12-1.20)	1.14 (1.04-1.25)	67.9%	0.014
No	8	1.17 (1.09-1.25)	1.17 (1.06-1.28)	7.8%	0.370
Study population age (year)					
≤ 10	7	1.16 (1.13-1.20)	1.17 (1.06-1.28)	64.5%	0.010
> 10	6	1.15 (1.07-1.23)	1.15 (1.07-1.23)	0%	0.692

### Heterogeneity and cumulative meta-analysis

The I^2^ value representing the overall between-study heterogeneity was 40.2% (*P* = 0.066), which was moderate and acceptable in the present meta-analysis. Clinical heterogeneity among subgroup studies is shown in Table 2. Additionally, statistical heterogeneity for a greater effect estimate from subgroups stratified by geographic area, population size, and publication year was low, varying from 0% to 21.7%. A meta-regression model was used to assess potential determinants of heterogeneity. Five factors (geographic area, population size, publication year, adjusted RR, and study population age) showed no statistical significance in either univariate analysis or the multivariate model (*P* >0.05, Table 3).

**Figure 4:**
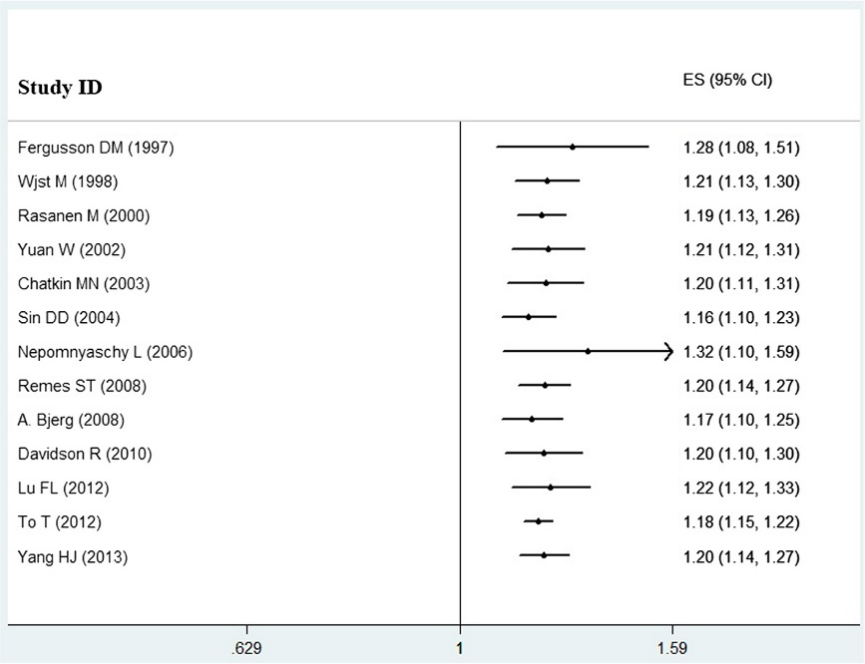
**Cumulative meta-analysis.** Each study was put into the pooled analysis one by one according to the publication year.

Cumulative meta-analyses of all of the studies showed that the estimates gradually became consistent, and the corresponding CIs narrowed down in the order of publication year (Figure 4). We also performed sensitivity analysis, and the pooled results did not appear to change.

**Table 3:** **Meta-regression analysis of the determinants of heterogeneity between study-specific effect estimates**

Determinants	Univariate analyses		
	Regression coefficient	95% CI	P value
Geographic area	-0.012	-0.155-0.131	0.853
Study population size	-0.00003	-0.0005-0.0004	0.874
Publication year	0.01	-0.011-0.031	0.330
Adjusted RR	0.035	-0.172-0.242	0.714
Study population age	-0.082	-0.308-0.144	0.440

### Publication bias analysis

**Figure 5:**
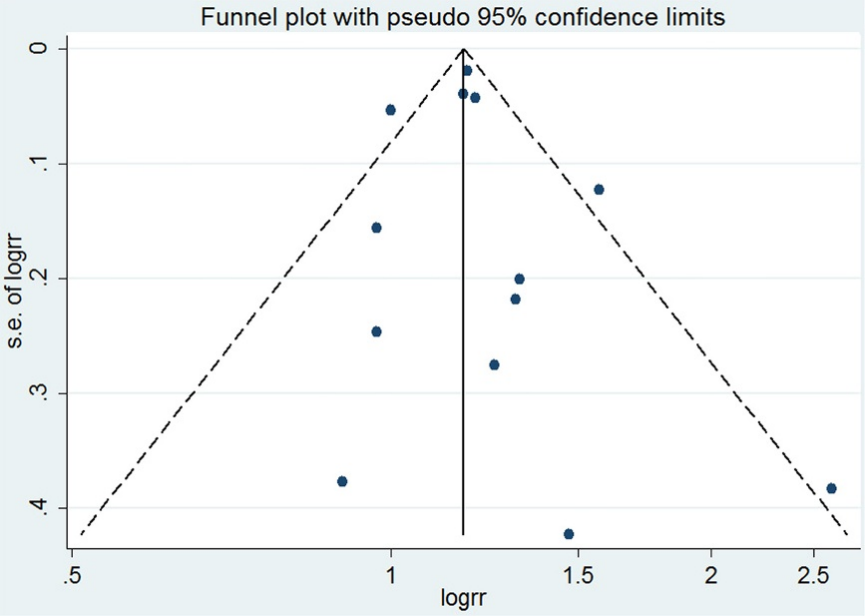
**Funnel plot of the effect estimates (log RR) by their SE (standard error of log RR).** The vertical line indicates the summary effect estimate and the corresponding pseudo 95% confidence limits converging as a function of the SE of the effect estimate.

We assessed the potential publication bias by using a funnel plot (Figure 5). The funnel plots did not show any remarkable asymmetry. Additionally, Egger’s and Begg’s tests showed no evident publication bias. P values for the two tests were 0.653 and 0.951, respectively.

## Discussion

In the present meta-analysis, 13 cohort studies and 1,105,703 subjects were included. By conducting a systematic review, we obtained an estimate that suggests that low birth weight among children increases the risk of future asthma. Children with low birth weight have an approximately 16% higher risk of asthma compared with those with normal birth weight. In stratified analyses, the effect of low birth weight on childhood asthma was strong, particularly in studies conducted in Europe, those with a small sample size, and those published recently. When the moderate heterogeneity was taken into account, meta-regression analysis did not show any significant determinants.

Several limitations of this meta-analysis should be considered. First, misclassification of exposure was a potential source of bias in this systematic review, especially because of acceptance of different definitions of low and normal birth weight. The acceptance of low birth weight, including less than 2500 g or 3000 g, varied for different cohorts. Second, when the adjusted estimates were unavailable, the calculated estimates without adjustment were likely to have potential confounding. Third, restriction of analysis to articles published in English is another potential limitation. Furthermore, the effect of low birth weight on childhood asthma in the identified studies was not the primary objective, possibly leading to missing relevant data that was not evident in the title or abstract.

Asthma is a serious global health problem, and its prevalence is increasing in most countries, especially among children [2]. Meta-analyses have shown that some adverse environmental factors could be associated with the development of asthma later in life. Exposure to wood dust, and prenatal or early life antibiotic exposure increase the risk of developing childhood asthma [5, 27]. Prenatal or early life probiotic administration reduces the risk of atopic sensitization in children, but may not reduce the risk of asthma [28]. Preterm infants have an increased risk of asthma compared with term neonates [29]. Moreover, children with a high birth weight or body weight later are at increased risk for future asthma [6]. The underlying biological mechanisms of this increased risk might include diet, gastro-oesophageal reflux, mechanical effects of obesity, atopy, and hormonal influences [6]. In addition to high birth weight, our meta-analysis further indicated that low birth weight was strongly correlated with the risk of childhood asthma.

Based on the present findings, low birth weight appears to be a significant risk factor for childhood asthma. If low birth weight can be considered as a modifiable risk factor, prevention of low birth weight could be associated with a decrease in the incidence of asthma. The purpose of this study was to evaluate the effect of low birth weight on the development of asthma later in life. In the present meta-analysis, we found that children with low birth weight had an approximately 16% higher risk of asthma. However, this association may be complicated by the fact that birth weight is not the only contributor to development of asthma. The effect of low birth weight on asthma is likely to be equally important compared with other risk factors, such as a family history of asthma and air pollution [3, 30].

The potential mechanisms of the association between low birth weight and an increased risk of childhood asthma are not fully understood. There is a wealth of epidemiological evidence that lower birth weight is strongly correlated with an increased risk of adult diseases, such as hypertension, type 2 diabetes mellitus, and cardiovascular disease [7, 31]. This phenomenon of fetal origins of adult disease is strongly associated with fetal insults to epigenetic modifications of genes [7, 32, 33]. Therefore, we speculate that epigenetic regulation may be involved in this mechanism of an increased risk of childhood asthma followed by low birth weight. Children with low birth weight could have varying degrees of problems with lung development, which would cause a greater sensitivity of an individual to external environmental stimuli and result in an increased risk of asthma. Additionally, children with low birth weight may be more likely to experience exposure to antibiotics, which has been previously shown to increase the risk of asthma [5].

In our analysis, significant differences were also observed across subgroups stratified by geographic area, population size, and publication year. Although the overall between-study heterogeneity was moderate, clinical heterogeneity among subgroup studies was low, indicating a greater effect estimate stratified by subgroup. Furthermore, these stratified factors were not found to be significant potential determinants in meta-regression. As in every meta-analysis, the quality of the individual studies may largely influence the review results. In the present analysis, all of the included studies possessed a high quality score. This heterogeneity in the present meta-analysis was acceptable.

## Conclusions

In summary, the weight of evidence shows that low birth weight significantly increases the risk of childhood asthma. The potential mechanisms underlying this association are not well understood. With regard to the relation between birth weight and asthma, special attention should be paid to the effect of different birth weights in children. Large-sample, well-designed, prospective cohort studies, should be performed in the future.
